# Crystal structure of a PP2A B56-BubR1 complex and its implications for PP2A substrate recruitment and localization

**DOI:** 10.1007/s13238-016-0283-4

**Published:** 2016-06-28

**Authors:** Jiao Wang, Zhizhi Wang, Tingting Yu, Huan Yang, David M. Virshup, Geert J. P. L. Kops, Sang Hyun Lee, Weihong Zhou, Xin Li, Wenqing Xu, Zihe Rao

**Affiliations:** College of Life Sciences, Nankai University, Tianjin, 30071 China; Department of Biological Structure, University of Washington, Seattle, WA 98195 USA; Program in Cancer and Stem Cell Biology, Duke-NUS Graduate Medical School, 8 College Road, Singapore, 169857 Singapore; Department of Pediatrics, Duke University Medical Center, Durham, NC 27710 USA; Molecular Cancer Research and Cancer Genomics Centre, and Department of Medical Oncology, UMC Utrecht, Universiteitsweg 100, 3584 CG Utrecht, The Netherlands; National Laboratory of Macromolecules, Institute of Biophysics, Chinese Academy of Sciences, Beijing, 100010 China

**Keywords:** PP2A, BubR1, kinetochore, cellular targeting, substrate recruitment

## Abstract

Protein phosphatase 2A (PP2A) accounts for the majority of total Ser/Thr phosphatase activities in most cell types and regulates many biological processes. PP2A holoenzymes contain a scaffold A subunit, a catalytic C subunit, and one of the regulatory/targeting B subunits. How the B subunit controls PP2A localization and substrate specificity, which is a crucial aspect of PP2A regulation, remains poorly understood. The kinetochore is a critical site for PP2A functioning, where PP2A orchestrates chromosome segregation through its interactions with BubR1. The PP2A-BubR1 interaction plays important roles in both spindle checkpoint silencing and stable microtubule-kinetochore attachment. Here we present the crystal structure of a PP2A B56-BubR1 complex, which demonstrates that a conserved BubR1 LxxIxE motif binds to the concave side of the B56 pseudo-HEAT repeats. The BubR1 motif binds to a groove formed between B56 HEAT repeats 3 and 4, which is quite distant from the B56 binding surface for PP2A catalytic C subunit and thus is unlikely to affect PP2A activity. In addition, the BubR1 binding site on B56 is far from the B56 binding site of shugoshin, another kinetochore PP2A-binding protein, and thus BubR1 and shugoshin can potentially interact with PP2A-B56 simultaneously. Our structural and biochemical analysis indicates that other proteins with the LxxIxE motif may also bind to the same PP2A B56 surface. Thus, our structure of the PP2A B56-BubR1 complex provides important insights into how the B56 subunit directs the recruitment of PP2A to specific targets.

**Electronic supplementary material:**

The online version of this article (doi:10.1007/s13238-016-0283-4) contains supplementary material, which is available to authorized users.

## **INTRODUCTION**

Protein phosphorylation controls many, if not most, critical biological processes among all clades of life. In eukaryotes, it is estimated about one third of all proteins may be reversibly phosphorylated (Cohen, 2000; Virshup and Shenolikar, 2009). Protein phosphatase 2A (PP2A) is a family of phosphatases that account for >50% of total Ser/Thr phosphatase activities in many cell types, and have a large number of substrates in the cell (Sangodkar et al., 2016; Virshup and Shenolikar, 2009; Wlodarchak and Xing, 2016). It is therefore crucial to understand how PP2A is localized to the right destination and recruits the correct substrate. PP2A predominantly exists as either a core dimeric complex consisting of the scaffolding A subunit and the catalytic C subunit, or a heterotrimeric holoenzyme that contains the AC core complex and a regulatory/targeting B subunit. There are at least 12 different B subunits in human PP2As that can be divided into four subfamilies (Sangodkar et al., 2016; Virshup and Shenolikar, 2009; Wlodarchak and Xing, 2016). It is generally assumed that the B subunit is responsible for the specificity of PP2A. In last several years, crystallographic and biochemical analysis have provided key insights into the structural basis of assembly and activity regulation of the PP2A holoenzymes (Cho et al., 2007; Cho and Xu, 2007; Xing et al., 2008; Xu et al., 2006). However, it remains poorly understood how the B subunit interacts with target/substrate proteins. To our knowledge, the crystal structure of a PP2A-shugoshin complex is so far the only PP2A-target complex structure available (Xu et al., 2009). Clearly, there is a critical gap in our understanding of PP2A substrate recognition and localization.

One of the key PP2A functions is to maintain genome stability and regulate the chromosome segregation process during cell division (Funabiki and Wynne, 2013; Wurzenberger and Gerlich, 2011). Accurate segregation of sister chromatids during mitosis requires the establishment of proper attachment of each chromosome to the spindle apparatus (Funabiki and Wynne, 2013; Sarangapani and Asbury, 2014). Unfavorable cell division conditions such as inappropriate kinetochore-microtubule connection can activate the spindle assembly checkpoint (SAC) signaling pathway, which prevents premature cell division by delaying the onset of anaphase (Musacchio, 2015). Both the proper chromosome-spindle connection and SAC are regulated by a number of crucial phosphorylation events catalyzed by protein kinases including Aurora B, Mps1 and Bub1 (Funabiki and Wynne, 2013; Musacchio, 2015). Recently, it has been shown that PP2A isoforms containing the B56 subfamily regulatory/targeting subunit (also known as B’ or PPP2R5, including α, β, γ, δ and ε isoforms) are required to counterbalance the phosphorylation homeostasis and prevent premature chromosome segregation (Espert et al., 2014; Foley et al., 2011; Nijenhuis et al., 2014; Suijkerbuijk et al., 2012; Xu et al., 2014). In particular, the B56 subfamily of PP2A localizes to the kinetochores/centromeres of unattached chromosomes through the interaction between the B56 subunit and BubR1 (Kruse et al., 2013; Suijkerbuijk et al., 2012; Xu et al., 2013).

The multi-domain pseudokinase BubR1 is a central component of the SAC and promotes chromosome congression at the kinetochore (Ditchfield et al., 2003; Lampson and Kapoor, 2005). It has been shown that a so-called ‘kinetochore attachment and regulatory domain’ (KARD) domain in the middle of BubR1 sequence is required for PP2A-B56 binding (Kruse et al., 2013; Suijkerbuijk et al., 2012; Xu et al., 2013). It has also been suggested that phosphorylation of three sites (Ser670, Ser676 and Thr680) in the KARD domain by Cdk1 and Plk1 can enhance the interaction between BubR1 and PP2A B56 subunit (Suijkerbuijk et al., 2012). However, how BubR1 interacts with PP2A B56 remains unclear. Here we present the crystal structure of a B56-BubR1 complex, which reveals the molecular basis of PP2A recruitment by BubR1 to the kinetochore. Our thermodynamic and GST-pulldown analyses of B56 and BubR1 mutant proteins also define the key B56 and BubR1 interface residues, and indicate a potential role of the specific KARD domain phosphorylation for this interaction. In addition, our work reveals an LxxIxE motif that is sufficient for PP2A B56 interaction, and suggests that this motif may be used by other PP2A-B56 binding proteins.

## **RESULTS**

### **Overall structure of the B56-BubR1 complex**

Previous studies indicated that a human BubR1 region containing residues 647–720 is sufficient for interacting with all human PP2A B56 isoforms including B56γ1 (Kruse et al., 2013; Suijkerbuijk et al., 2012; Xu et al., 2013). To reveal the potential contribution from the phosphorylation of the three BubR1 KARD domain residues, we generated a BubR1 mutant with all three sites mutated to aspartic acid (S670D/S676D/T680D), which was previously used to mimic BubR1 phosphorylation (Suijkerbuijk et al., 2012). For convenience, we refer to this triple mutant as BubR1-3D. We found that the B56/BubR1(647–720)-3D complex is stable during size exclusion chromatography (SEC) (Fig. S1) and GST-pulldown (See below).

**Figure 1:**
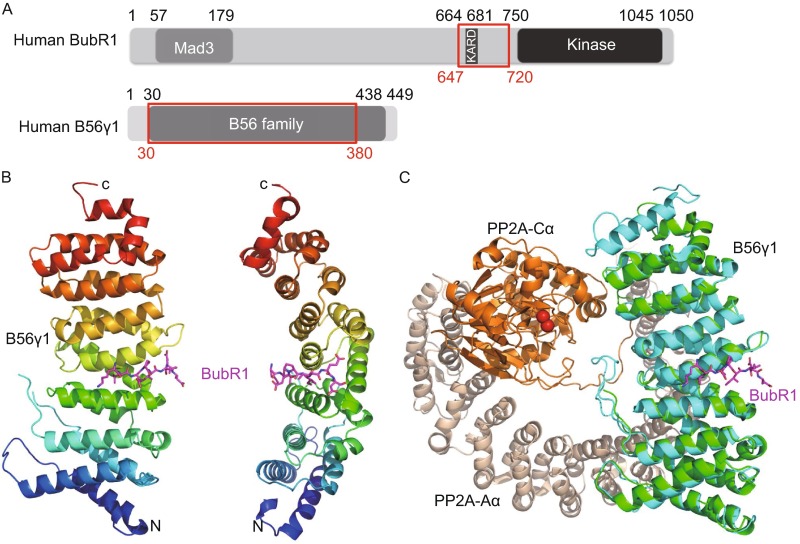
**Overall structure of the B56-BubR1 complex**. (A) Schematic representations of domain structures of human BubR1 and PP2A B56γ1 proteins. Numbers above indicate amino acid position based on the sequence of human BubR1 and B56γ1. The fragments used for crystallization are indicated by red boxes. (B) Overall structure of the B56γ1(30–380)/BubR1(647–720)-3D complex, in two orthogonal views. (C) Superposition of the B56γ1-BubR1 structure with that of the Aα-B56γ1-Cα PP2A holoenzyme (PDB code: 2IAE). The red spheres are the two Mn^2+^ ions in the PP2A active site.

To provide the structural basis for understanding how PP2A is recruited to the kinetochore through its interaction with BubR1, we determined the crystal structure of human PP2A B56γ1(30–380) in complex with human BubR1(647–720)-3D at 2.35 resolution (Table S1). B56γ1(30–380), B56γ1 for short hereafter, forms the ordered region of the conserved B56 domain (Fig. 1A). Similar to the corresponding B56 structures alone and in the PP2A holoenzyme (Cho and Xu, 2007; Magnusdottir et al., 2009; Xu et al., 2006; Xu et al., 2009), B56γ1 in our complex contains seven and half HEAT repeats and forms a solenoid shape. There are two B56γ1-BubR1 complexes in each asymmetric unit, with almost identical structures (r.m.s.d. of 0.277 Å for 2281 non-hydrogen atoms). Although BubR1(647–720)-3D contains 74 residues, we were only able to visualize electron densities for 8 residues in a groove formed between the HEAT repeats 3 and 4 (Figs. 1B and S2). Unsolved BubR1 residues are expected to be disordered and not involved in B56γ1 binding. These eight residues are KLDPIIED, corresponding to BubR1 residues 668–675, with phosphor-Ser670 replaced by Asp670.

When our B56γ1-BubR1 complex structure is superimposed on that of the A-B56γ1-C PP2A trimeric holoenzyme, it is clear that the visible BubR1 segment is located in the B56 surface groove that is opposite to the face that interacts with the PP2A catalytic C subunit (Fig. 1C). In addition, the BubR1 binding site on B56γ1 does not overlap with that of the PP2A scaffold A subunit. Therefore BubR1 should not affect PP2A enzymatic activity and should not interfere with the PP2A holoenzyme assembly. Furthermore, giving the spatial position of BubR1 Ser670 and direction of the BubR1 peptide binds to the B56 surface, none of the three BubR1 KARD domain phosphorylation sites (Ser670, Ser676 and Thr680) can be accessed by the PP2A C active site of the same PP2A holoenzyme (*in cis*). These observations provide new mechanistic insights into the B56-BubR1 interaction in PP2A localization and chromosome segregation during cell division.

### **The interaction interface in the B56-BubR1 complex**

**Figure 2:**
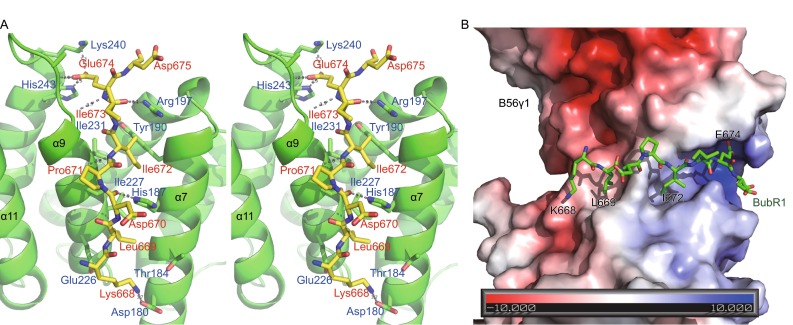
**Details of the B56-BubR1 interface**. (A) Stereo view of the interface interactions. BubR1 and B56 residues are labelled in red and blue, respectively. (B) The BubR1 KARD domain structure (in sticks) is laid on top of electrostatic surface representation of B56γ1.

Among these eight BubR1 residues visible in the electron density map, four residues (Lys668, Leu669, Ile672 and Glu674) form sidechain-mediated interactions with B56γ1 (Fig. 2). Leu669 resides in a pocket formed by B56γ1 sidechains of His187, Ser173, Ile227, Thr184 and Glu226; whereas the sidechain of BubR1 Ile672 is located in a hydrophobic pocket formed by B56γ1 residues His187, Ile231, Tyr190 and Ser230. The carboxyl group of BubR1 Glu674 settles in a positively charged pocket, and its sidechain forms hydrogen bonds with B56 His243 sidechain and the mainchain amine group of B56 Ala236, respectively. In addition, Glu674 also forms a salt bridge with the B56γ1 Lys240 sidechain amine group (Fig. 2). In contrast to the clear sidechain densities for BubR1 Leu669, Ile672 and Glu674, the sidechain density for Lys668 is not as well-defined (Fig. S2), but appears to form a charge-charge interaction with the B56γ1 sidechain Asp180. Other than these four residues, the four other sidechains of visible residues extend into the solvent and make only marginal contacts with B56γ1.

In addition to the sidechain interactions, the mainchain of BubR1 also interacts with B56γ1 and helps to guide the docking of the BubR1 peptide in the B56γ1 groove, predominantly by interacting with two B56γ1 residues, His187 and Arg197. The His187 sidechain forms a hydrogen bond with the carbonyl of BubR1 Asp670 (mimicking the phosphorylated Ser670), whereas the Arg197 sidechain forms a hydrogen bond with the carbonyl of BubR1 Ile673. Furthermore, the mainchain amine groups of BubR1 Ile672 and Glu674 form hydrogen bonds with mainchain carbonyl groups of B56γ1 Ser230 and Gly234, respectively.

**Figure 3:**
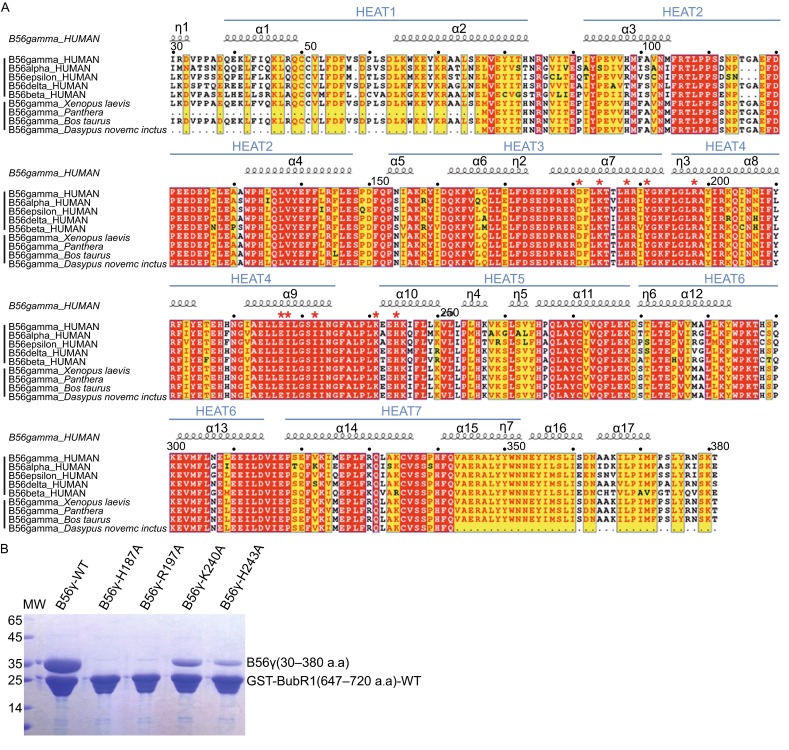
**Sequence alignment of B56**γ**1 and mutagenesis of B56**γ**1**. (A) B56 sequence alignment. The upper half shows the alignment of the five different human B56 isoforms, whereas the lower half demonstrates the sequence conservation of B56γ from different organisms. Numbers above indicate amino acid position based on the sequence of human B56γ1. B56γ has three isoforms with different C-terminal domains. Only B56γ1 is shown here. The strictly conserved residues are in white letters with red background and the conserved residues are in red letters with yellow background. Residues involved in BubR1 interaction are indicated with red asterisks. (B) Pulldown of mutant B56γ1 proteins by WT GST-BubR1(647–720).

It should be noted that all BubR1-interacting residues are conserved in different B56 isoforms in human (Fig. 3A), consistent with earlier observation that all B56 forms can interact with BubR1 (Kruse et al., 2013; Suijkerbuijk et al., 2012; Xu et al., 2013).

### **Mutagenesis analysis of B56 interface residues validate the BubR1 binding site**

To validate the crystallographic observations and evaluate the significance of individual interaction, we performed mutagenesis analysis on both sides of the PP2A B56 and BubR1 interface. Mutant B56γ1 and BubR1 proteins were analyzed using both GST-pulldown and isothermal titration calorimetry (ITC) assays. First of all, our GST-pulldown assay demonstrated that GST-BubR1(647–720) can pulldown B56γ1, likely in 1:1 molar ratio (Fig. 3B). This is consistent with our observation that purified B56γ1 and BubR1-3D co-migrate in SEC (Fig. S1). Our ITC analysis showed that binding between B56γ1 and BubR1(647–720) has a *K*d of 2.9 μmol/L (Table 1 and Fig. S3). Among protein complexes that demonstrate SEC co-migration and stoichiometric GST-pulldown, this *K*d value seems quite high. It is possible that the B56-BubR1 interactions have a relatively low off rate that can kinetically trap both proteins in the complex during SEC and pulldown.

**Table 1:** **Summary of ITC analysis of the PP2A B56-BubR1 interaction**

**Well protein**	**Titration**	**N** **(Sites)**	**ΔH** **(kcal/mol)**	**TΔS** **(kcal/mol)**	***K*** **d** **(μmol/L)**
B56γ-WT	BubR1-WT	1.08 ± 0.03	−11.57 ± 0.42	−4.25	2.91 ± 0.50
	BubR1-3D	1.02 ± 0.02	−28.36 ± 0.83	−20.87	2.27 ± 0.37
	BubR1-K667A	1.03 ± 0.03	−8.44 ± 0.33	−1.04	2.52 ± 0.37
	BubR1-K668A	0.99 ± 0.08	−10.49 ± 1.11	−3.56	5.65 ± 1.00
	BubR1-L669A	N/B	N/B	N/B	N/B
	BubR1-S670A	1.06 ± 0.05	−7.29 ± 0.50	−0.16	2.34 ± 0.63
	BubR1-P671A	1.10 ± 0.10	−7.51 ± 0.88	−0.38	4.08 ± 1.44
	BubR1-I672A	N/B	N/B	N/B	N/B
	BubR1-I673A	1.01 ± 0.02	−7.55 ± 0.21	−0.43	4.12 ± 0.33
	BubR1-E674A	N/B	N/B	N/B	N/B
	BubR1-D675A	1.09 ± 0.03	−12.64 ± 0.55	−5.12	2.06 ± 0.41
	BubR1-S676A	1.07 ± 0.04	−10.94 ± 0.64	−3.82	4.17 ± 0.75
	BubR1-R677A	1.06 ± 0.11	−6.08 ± 0.83	−0.84	5.85 ± 1.72
	BubR1-AAAA	1.02 ± 0.05	−9.74 ± 0.64	−3.15	10.56 ± 1.72
B56γ-H187A	BubR1-WT	N/B	N/B	N/B	N/B
B56γ-R197A		N/B	N/B	N/B	N/B
B56γ-K240A		0.97 ± 0.33	−12.04 ± 5.25	−6.01	27.86 ± 12.41
B56γ-H243A		1.0*	−30.47 ± 8.27	−25.03	80.00 ± 34.82

All the B56γ1 proteins listed here are a truncation form of human B56γ1 (residues 30–380), whereas all BubR1 proteins listed contain human BubR1 residues 647–720

N/B: no detectable binding

* The N number was fixed at 1.00 for this calculation, since the *K*d value is significantly higher than the B56γ1 protein concentration used

On the PP2A B56 side, we generated missense mutants at four surface residues that are involved in BubR1 interaction: His187, Arg197, Lys240 and His243. Our GST-pulldown results clearly show that either H187A or R197A mutation completely disrupted the B56-BubR1 interaction, whereas both K240A and H243A mutations significantly reduced the binding affinities with B56γ1. Our ITC data are fully consistent with the GST-pulldown results. There is no detectable binding between BubR1 and B56γ1 H187A or R197A mutant; whereas for B56 K240A and H243A mutants, the *K*d values of the interactions increased to 28 µmol/L and 80 µmol/L, respectively (Table 1). These results provide a clear validation for the BubR1 binding site on B56γ1 that we observed in our crystal structure.

### **Mutagenesis analysis of the B56-binding motif of BubR1 reveals a core B56 binding motif**

To understand if the short BubR1 region observed in our B56γ1-BubR1 complex is sufficient for PP2A-B56 binding, we performed ITC analysis of the interaction between B56γ1 and a short 15 residue peptide BubR1 (KKL**S**PIIED**S**REA**T**H; corresponding to WT BubR1 residues 667–681). The eight BubR1 residues visible in the electron density are underlined, whereas the three potential phosphorylation sites are in bold. With the same B56γ1, the *K*d values for this 15-mer and the 74-residue WT BubR1(647–720) are almost identical (2.8 µmol/L vs. 2.9 µmol/L; Tables 1 and 2), strongly indicating that the short 15-mer segment of BubR1 is solely responsible for B56 binding.

**Table 2:** **Summary of ITC analysis of the interactions between PP2A B56**γ**1 and phosphorylated or phosphor-mimicking BubR1 proteins/peptides**

**Well protein**	**Titration**	**N** **(Sites)**	**ΔH** **(kcal/mol)**	**TΔS** **(kcal/mol)**	***K*** **d** **(μmol/L)**
B56γ-WT	BubR1-WT	1.08 ± 0.03	−11.57 ± 0.42	−4.25	2.91 ± 0.50
	BubR1-3D	1.02 ± 0.02	−28.36 ± 0.83	−20.87	2.27 ± 0.37
	BubR1-SDD	1.03 ± 0.02	−10.40 ± 0.22	−2.98	2.40 ± 0.25
	BubR1-DSD	1.19 ± 0.01	−21.50 ± 0.35	−14.25	3.37 ± 0.29
	BubR1-DDT	1.12 ± 0.01	−19.42 ± 0.27	−12.11	3.09 ± 0.22
	BubR1-SDT	1.14 ± 0.02	−11.28 ± 0.23	−4.28	5.24 ± 0.37
	BubR1-DST	1.14 ± 0.02	−9.16 ± 0.27	−1.71	2.33 ± 0.37
	BubR1-SSD	1.16 ± 0.03	−14.78 ± 0.53	−7.72	4.61 ± 0.52
	KKLSPIIEDSREATH	1.02 ± 0.02	−12.06 ± 0.34	−4.71	2.80 ± 0.30
	KKL_p_SPIIEDSREATH	1.02 ± 0.01	−9.55 ± 0.20	−0.82	0.25 ± 0.06
	KKLSPIIED_p_SREATH	0.99 ± 0.02	−11.08 ± 0.31	−2.92	0.69 ± 0.11
	KKLSPIIED_p_SREA_p_TH	1.02 ± 0.01	−10.73 ± 0.18	−2.41	0.52 ± 0.06
B56γ-R188A	KKL_p_SPIIEDSREATH	1.07 ± 0.03	−6.75 ± 0.27	−0.84	1.86 ± 0.30
B56γ-R201A	KKLSPIIED_p_SREATH	N/B	N/B	N/B	N/B

B56γ1-WT indicates wild-type human B56γ1 (residues 30–380). All BubR1 proteins listed contain human BubR1 residues 647–720, except for the 15-mer BubR1 peptides (corresponding to BubR1 residues 667–681), in which pS and pT represent phosphor-serine and phosphor-threonine, respectively

N/B: no detectable binding

**Figure 4:**
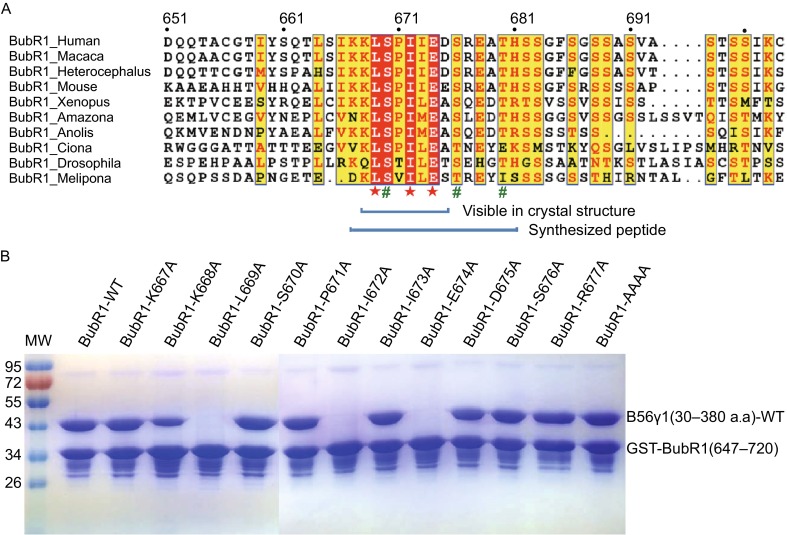
**Sequence alignment of BubR1 and mutagenesis analysis of the B56**γ**1-BubR1 interface**. (A) Sequence alignment of the KARD domain of BubR1. BubR1 residues essential for B56 binding are labelled with red stars, whereas phosphorylation sites are labeled green pound signs. (B) GST-pulldown of B56γ1 by mutant GST-BubR1(647–720) proteins. BubR1-AAAA represents GST-tagged BubR1(647–720; E678A/T680A/H681A).

To understand which residues in this WT 15-mer sequence are important for B56 binding, we generated 11 single-missense Ala mutants and one mutant with simultaneous mutation of the last four residues in this 15-mer sequence. Our GST-pulldown data showed that three BubR1 mutations, L669A, I672A and E674A, completely disrupted the BubR1-B56 interaction, while the BubR1 K668A mutation slightly reduced the B56 binding (Fig. 4B). There was no significant change in GST-pulldown for all 8 of the remaining BubR1 mutations (Fig. 4B). Our ITC data also show that the L669A, I672A and E674A mutations abolished the BubR1-B56 interaction, although the K668A mutation did not lead to any dramatic *K*d change. All these binding analyses are fully consistent with our crystal structure, in which only these four sidechains are involved in B56 binding, and Leu669, Ile672 and Glu674 bind tightly to their respective pockets. These results also suggest that any peptide with an LxxIxE motif in a flexible region of a protein may bind to PP2A B56 in a manner similar to BubR1 (a buried or semi-buried LxxIxE motif may not be compatible with the B56 binding).

### **Analysis of the effect of BubR1 KARD domain phosphorylation on B56 binding**

Among the three potential BubR1 KARD domain phosphorylation sites (Ser670, Ser676 and Thr680), only Ser670 is within the visible KL**S**^670^PIIED segment. It was surprising that the sidechain of Asp670 that mimics the phosphoserine face solvent and does not make significant contact with B56γ1. To test if any aspartate mutation in the BubR1 KARD domain can mimic phosphor-serine/threonine with respect to direct B56 binding, we generated all 8 mutant combinations at these three sites and measured that binding affinities with B56γ1 using ITC. It appears that all these 8 BubR1 forms, including WT and the 3D mutant, have very comparable *K*d’s with B56γ1 (Table 2), indicating that aspartate cannot mimic the effect of phosphor-serine/threonine in any of these three sites.

To explore whether phosphorylation at these three BubR1 sites can enhance the BubR1-B56 interaction, we performed ITC analysis of the interaction between B56γ1 and three chemically-synthesized 15-mer BubR1 phospho-peptides (Table 2). Our ITC results demonstrate that phosphorylation of Ser670 can enhance the binding by ~11 fold, and phosphorylation of Ser676 can also strengthen the binding by ~4 fold, while Thr680 phosphorylation may not be significant. The stronger effect of phosphoserine than aspartate can be explained by charge difference and the longer sidechain for phosphoserine. To provide a structural explanation for enhanced binding for pSer670 and pSer676, we did structural modeling by replacing the aspartate sidechains with phosphoserine. It appears that the BubR1 pSer670 phosphate group may enhance the B56-BubR1 binding by interacting with a neighboring B56γ1 Arg188 (Fig. S5), while the BubR1 pSer676 phosphate group may reach a positively charged pocket and interact with B56 Arg201 that Asp676 cannot reach (Fig. S5). In support of this model, our ITC analysis showed that the B56γ1 R188A mutation mostly canceled the enhancing effect of Ser670 phosphorylation in the BubR1 pSer670-phosphopeptide (with *K*d’s changed from 0.25 µmol/L to 1.86 µmol/L; Table 2 and Fig. S5). B56γ1 R201A mutation completely abolished the binding of the BubR1 pSer676-phosphopeptide (Table 2 and Fig. S5), likely because mutation of the semi-buried B56 Arg201 induced major local conformational changes, which in turn affected the interaction of the critical BubR1 Glu674 residue in positively charged pocket (Figs. 2 and S5).

## **DISCUSSION**

Among all PP2A forms, the PP2A B56 family plays a critical role in cancer biology, and is involved in dephosphorylation and regulation of a number of critical oncoproteins and tumor suppressors in the cell, including p53, myc and Wnt pathway components including β-catenin and APC (Arnold and Sears, 2006; Gao et al., 2002; Li et al., 2007; Li et al., 2001; Seeling et al., 1999; Virshup and Shenolikar, 2009). How B56 recruits PP2A to the right cellular locations to recognize its large number of substrates remains a critical unanswered question. Here we demonstrate that a short peptide with the LxxIxE motif can bind specifically to B56. Since the LxxIxE motif can be found in a large number of protein sequences, with many of them potentially having this motif in a flexible region, our work may reveal a common signal for recruitment of the B56 family of PP2A.

BubR1 is hyperphosphorylated during mitosis, and phosphorylation of the BubR1 KARD domain by Cdk1 and Plk1 has been proposed to play a key regulatory role for BubR1 in establishing error-free kinetochore-microtubule attachments and alignment of duplicated chromosomes at the metaphase plate. In particular, Cdk1 has been shown to phosphorylate Ser670 (Elowe et al., 2007) in response to lack of kinetochore-microtubule attachment, while Plk1 phosphorylates Ser676 (Elowe et al., 2007) and Thr680 (Suijkerbuijk et al., 2012) in response to lack of tension. Notably, it was shown that the triple mutation of three phosphorylation sites to Asp can somehow mimic the phosphorylation of these three sites in cells (Suijkerbuijk et al., 2012). Our ITC data demonstrate that Asp mutants cannot mimic phosphoserine for B56 binding *in vitro*, and consistent with a previous study (Kruse et al., 2013), illustrate that Ser670/676 phosphorylation can indeed promote the direct interaction between B56 and BubR1, while Thr680 phosphorylation may not be significant. Although co-crystallization of B56 with BubR1 phosphopeptides has not been successful so far, our current crystal structure provides clues to understand how these BubR1 KARD phosphorylation events promote PP2A-B56 recruitment to the kinetochore via interaction with BubR1 (Fig. S5). Since Thr680 contributes to KARD function and KARD-3D rescues BubR1 depletions (Suijkerbuijk et al., 2012), there may be an additional indirect contribution of KARD phosphorylation to PP2A binding and/or function at kinetochores.

The way the BubR1 KARD domain docks on PP2A-B56 suggests that BubR1 does not interfere with PP2A enzymatic activity and assembly, and that the BubR1 KARD domain *per se* is unlikely to be a substrate of the same PP2A molecule to which BubR1 binds. Therefore, dephosphorylation of the BubR1 KARD domain, which may be required for eventual release of PP2A from BubR1, would rely on another phosphatase molecule. Notably, PP2A B56 antagonizes Aurora B to promote PP1 recruitment to kinetochore (Nijenhuis et al., 2014). PP1 in turn silences the SAC and delocalizes PP2A B56 from kinetochore. Thus, this release of PP2A from BubR1 may be also achieved by PP1. Alternatively, BubR1 and PP2A may be released together from kinetochore via PP1-mediated dephosphorylation of BubR1 kinetochore docking site (Nijenhuis et al., 2014) that allows the cell to enter the anaphase.

**Figure 5:**
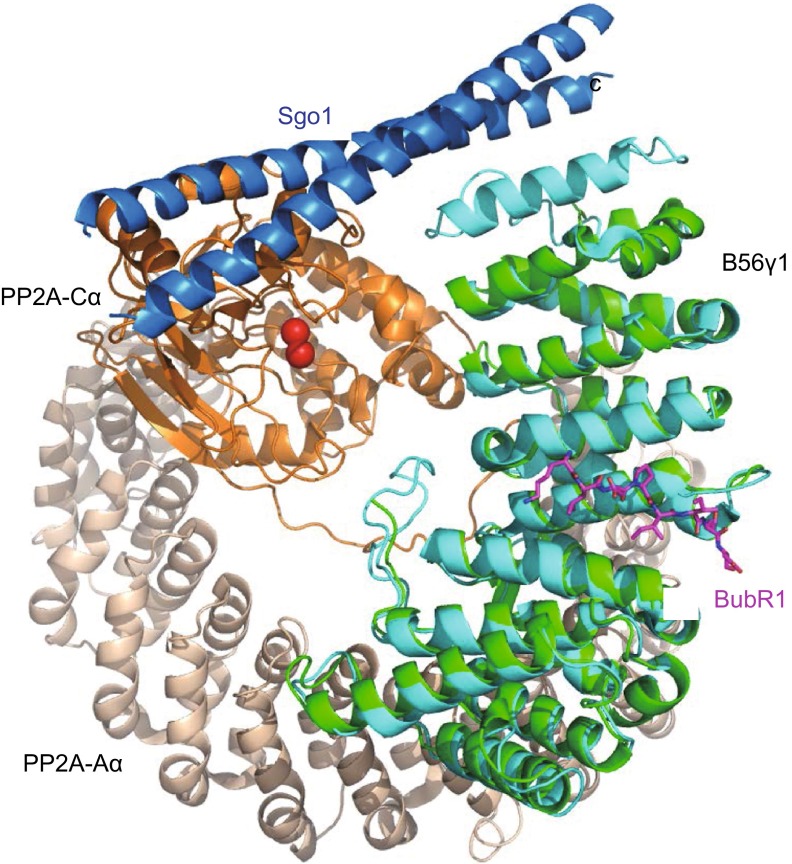
**Superposition of the B56-BubR1 complex with the PP2A-shugoshin complex**. The Aα-B56γ1-Cα PP2A/Sgo1 complex structure (PDB code: 3FGA) is superimposed to the current B56γ1-BubR1 complex structure, based on the common B56γ1 subunit. The Sgo1 coiled-coil domain homodimer, which is responsible for PP2A binding, is in blue color.

It is interesting to note that in addition to BubR1, another protein, shugoshin (Sgo1) can also recruit B56-bound PP2A to the inner centromere (Kitajima et al., 2004; Kitajima et al., 2006; Liu et al., 2013b; Riedel et al., 2006; Tang et al., 2006; Hara et al., 2014). Superposition of the PP2A-Sgo1 complex structure with the B56γ1-BubR1 complex structure demonstrates that Sgo1 and BubR1 bind to two far-separated surfaces of B56γ1 (Fig. 5). Although the biological significance of this observation awaits future investigation, as a pool of Sgo1 is also found at the kinetochore (Liu et al., 2013a), our results indicate that Sgo1 and BubR1 may bind to the same PP2A B56 molecules simultaneously. Alternatively, these different modes of interaction may be required for a stable complex formation of Sgo1 with PP2A B56-Sgo1 at the centromere in order to protect cohesin from the beginning until the end of mitosis. In contrast, PP2A B56-BubR1 complexes formed via the LxxIxE motif may be more transient in order to fine-tune the balance between stabilization and destabilization of kinetochore-microtubule attachment. Thus, this transient phosphorylation-dependent interaction between PP2A B56 and BubR1 may allow efficient error-correction and error-free segregation of chromosomes. Interestingly, in HeLa cells, B56γ and B56δ preferentially localize to kinetochores, whereas B56α, B56β and B56ε appear to localize to centromeres (Nijenhuis et al., 2014). The relative abundance, binding affinity and mode of interaction of the B56 isoforms to BubR1 and Sgo1 in a given cell type may determine the preferential localization of distinct PP2A B56 holoenzyme to either centromere or kinetochore. How preferential localization of B56 isoforms is determined and its biological significance is clearly a matter of interest for future studies.

## **MATERIALS AND METHODS**

### **Expression, purification and crystallization of the B56**γ1 **and BubR1-3D complex**

The truncated domains of human B56γ1 (residues 30–380) and the human BubR1-3D mutant (residues 647–720) were separately cloned into the pCool vector between *Nde*I/*Not*I sites with a TEV protease cleavage site located between the GST tag and the target protein. These two recombinant plasmids were transformed into *E. coli* BL21 (DE3) cells (Novagen). The cells were grown in LB medium containing 50 mg/L ampicillin at 37°C. When the OD_600_ of the culture reached 0.6, 0.1 mmol/L isopropyl β-D-thiogalactopyranoside (IPTG) was added to induce the protein expression for 16 h at 16°C. The cells were then harvested by centrifugation. The pellets were separately re-suspended in 20 mmol/L Tris-HCl pH 8.0, 500 mmol/L NaCl and 5 mmol/L DTT and subsequently disrupted by sonication. The GST fusion proteins were first purified by Glutathione Sepharose 4B beads (GE Healthcare) and the GST tag was removed by TEV protease at 4°C overnight. The proteins were further purified separately by 1 mL Hitrap Q HP column (GE Healthcare). Then the purified B56γ1 and BubR1-3D proteins were mixed with a molar ratio 1:1.2 in 20 mmol/L Tris-HCl pH 8.0, 250 mmol/L NaCl and 5 mmol/L DTT and incubated at 4°C overnight. The excessive BubR1-3D protein was removed by Superdex 200 10/300 GL column (GE Healthcare) in 20 mmol/L Tris-HCl pH 8.0, 250 mmol/L NaCl and 5 mmol/L DTT. The purity of complex samples was verified using SDS-PAGE stained with Coomassie Brilliant Blue and then concentrated to 8 mg/mL in 20 mmol/L Tris-HCl pH 8.0, 250 mmol/L NaCl, 5 mmol/L DTT for crystallization. The sitting drop vapor diffusion method was used to prepare crystals for the B56γ1 and BubR1-3D protein complex. 1 µL of protein complex was mixed with 1 µL of reservoir solution containing 0.1 mol/L HEPES pH 7.5 and 20% PEG 3350. Crystals grew to their full size in about 10 days at 4°C. The crystals were directly cryo-cooled in liquid nitrogen.

### **Data collection and structure determination**

The crystal of the B56γ1/BubR1-3D complex diffracted to 2.35 Å resolution in Beamline 17U of Shanghai Synchrotron Radiation Facility. The HKL2000 package (Otwinowski et al., 1997) was used for data processing. The structure was determined by molecular replacement with the program PHASER (McCoy et al., 2007), using the crystal structure of B56γ1 (PDB: 2JAK) as a template. Two molecules of B56γ1 were found in one asymmetric unit. The complex model was improved using iterative cycles of manual rebuilding with COOT (Emsley et al., 2010) and refinement with Refmac5 in CCP4 (Winn et al., 2011). TLS were used throughout refinement (Winn et al., 2003). The final refinement statistics were summarized in Table S1. The structural figures were drawn using Pymol (DeLano and Brunger, 1994). The electrostatic potential surfaces shown were generated by the APBS tool in Pymol.

### **Mutagenesis and phosphopeptides**

All the mutagenesis of B56γ1 and BubR1 mentioned in this paper were made by Fast mutagenesis system kit (Transgen) and purified by the same way as wild type. All GST-fusion proteins including B56γ (wild type and variants) and BubR1 (wild type and variants) were purified in the same way as described above. The 15-mer phosphopeptides of BubR1 were synthesized (AuGCT Company, China).

### **GST pulldown assay**

All the GST-tagged proteins including B56γ1 (wild type and variants) and BubR1 (wild type and variants) were purified the same way described previously without TEV cleavage. The purified GST-fusion proteins were mixed with untagged protein with a molar ratio 1:3 in 20 mmol/L Tris-HCl pH 8.0 and 200 mmol/L NaCl at RT for 1 h. Glutathione Sepharose 4B beads (GE Healthcare) were added to the protein mixture and incubated at RT for 1 h. The unbound proteins were removed by washing with buffer (20 mmol/L Tris-HCl pH 8.0 and 200 mmol/L NaCl) for 5 times. The bound proteins were separated by SDS-PAGE and stained by Coomassie Brilliant Blue.

### **Isothermal titration calorimetry (ITC)**

The binding affinities of BubR1 (wild type and variants) for B56γ1 (wild type and variants) were assayed on an iTC200 microcalorimeter (MicroCal) at room temperature. B56γ1 samples (wild type and variants) were placed in the reaction cell at a concentration of 0.03 mmol/L in the buffer containing 20 mmol/L Tris-HCl pH 8.0 and 200 mmol/L NaCl. BubR1 (wild type, variants and the phosphopeptide) at a concentration of 0.7 mmol/L were injected in 1.5 µL quantities every 120 s for a total of 20 injections into B56γ1 samples (wild type and variants). Data were fit with a one-site binding model using Origin 7.0.

## Electronic supplementary material

Below is the link to the electronic supplementary material.

Supplementary material 1 (PDF 12275 kb)
