# Supplementary material for: Crystal structure of a PP2A B56-BubR1 complex and its implications for PP2A substrate recruitment and localization
Source: Protein Cell. 2016 Jun 28;7(7):516–26. doi: 10.1007/s13238-016-0283-4 (PMC4930772; doi:10.1007/s13238-016-0283-4)
Supplement: Supplementary file 1 — Supplementary material 1 (PDF 12275 kb) [file 13238_2016_283_MOESM1_ESM.pdf]

**Table S1. Diffraction Data and Refinement**	
Dataset	B56γ1/BubR1-3D complex
Space group	$P2_12_12_1$
Cell dimensions	a=87.02Å, b=95.59Å, c=167.37Å, α=β=γ=90.00°
Cell content	2 protein complexes/AU
Solvent content	65.74%
**Data Collection**	
Wavelength	0.98Å
Resolution	50.00Å - 2.35Å (2.39Å - 2.35Å)
Unique reflections	54953
Rsym	8.7% (45.5%)
<I>/<SIGI>	24.6 (6.0)
Completeness	100.0% (100.0%)
$CC_{1/2}$	(0.947)
Redundancy	10.5 (11.0)
**Refinement**	
Rwork/Rfree	18.0% / 20.4%
Average B factor	32.63Å^2
Rmsd bond distance	0.007Å
Rmsd angle angle	1.083°
Ramachandran favored	99.4%
Ramachandran allowed	0.6%
Protein residues	682
Water	240

AU, asymmetric unit; Rmsd, root-mean-square deviation. Values in parentheses indicate the corresponding statistics in the highest-resolution shell. $CC_{1/2}$ is the correlation coefficient between symmetry-related intensities taken from random halves of the dataset.

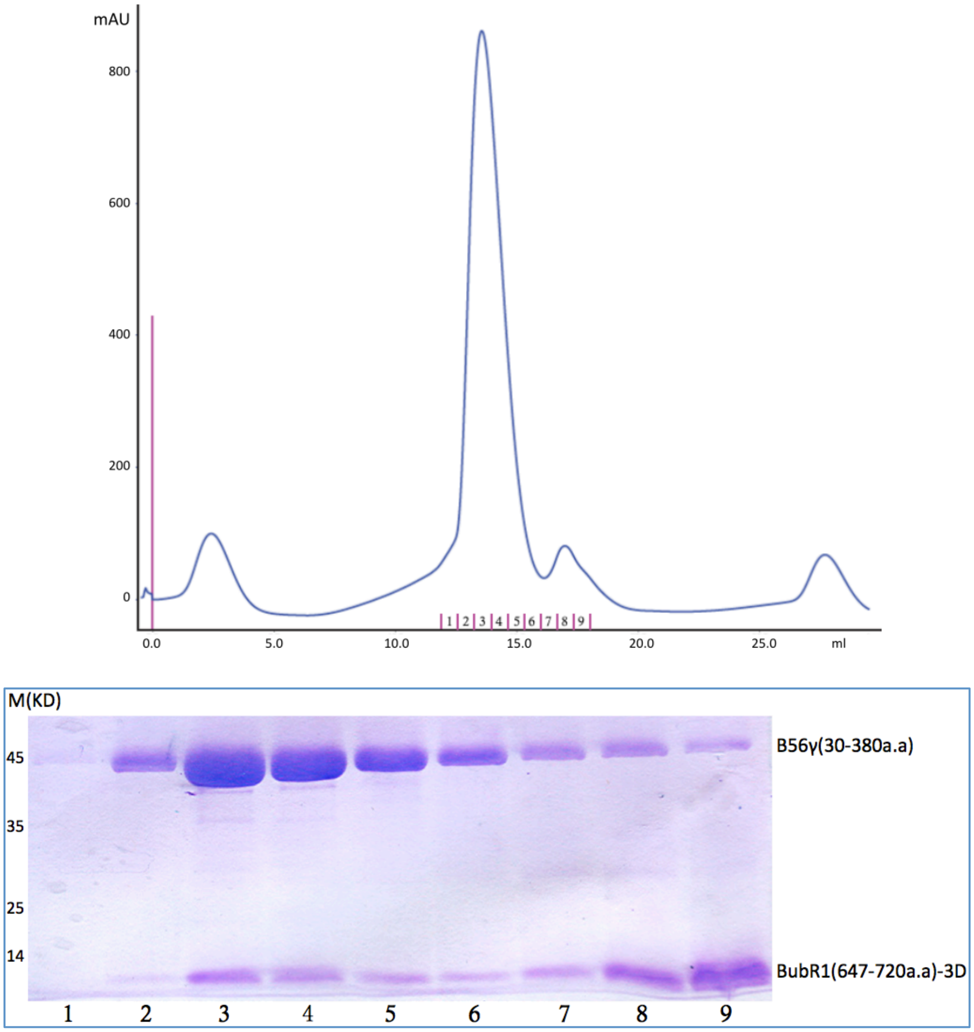

Figure S1. Co-purification of the B56γ1(30-380)-BubR1(647-720) complex. (Top) A typical SEC chromatogram of the B56γ1-BubR1 complex purification is shown. (Bottom) Fractions corresponding to the peak are analyzed by SDS-PAGE and Coomassie Blue staining.

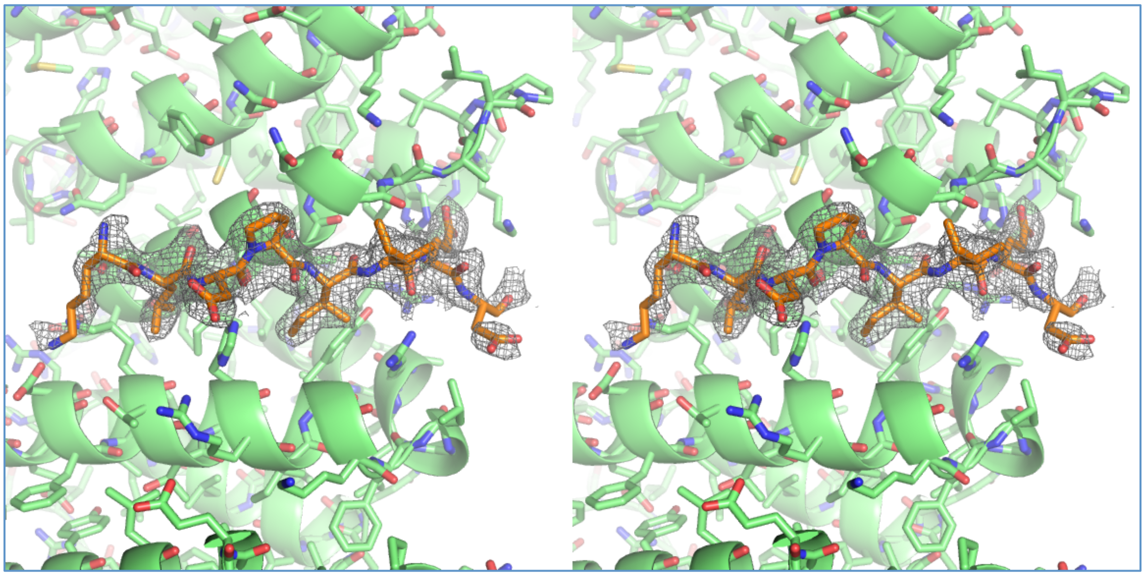

Figure S2. Stereo view of the 2Fo-Fc electron density map contoured at 1.0σ. The composite simulated annealing omit maps were calculated to reduce the effects of model bias.

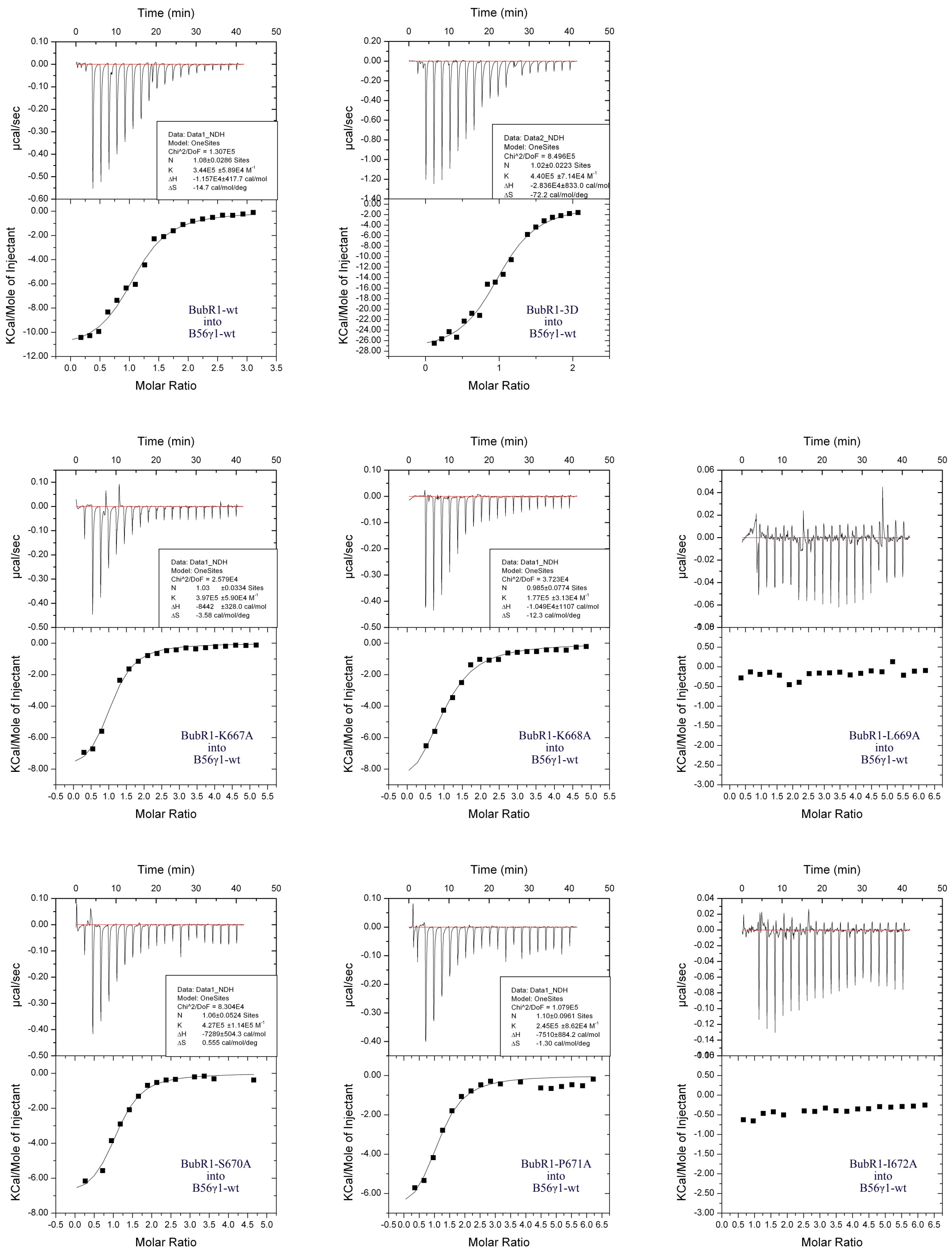

This figure is continued in the next page.

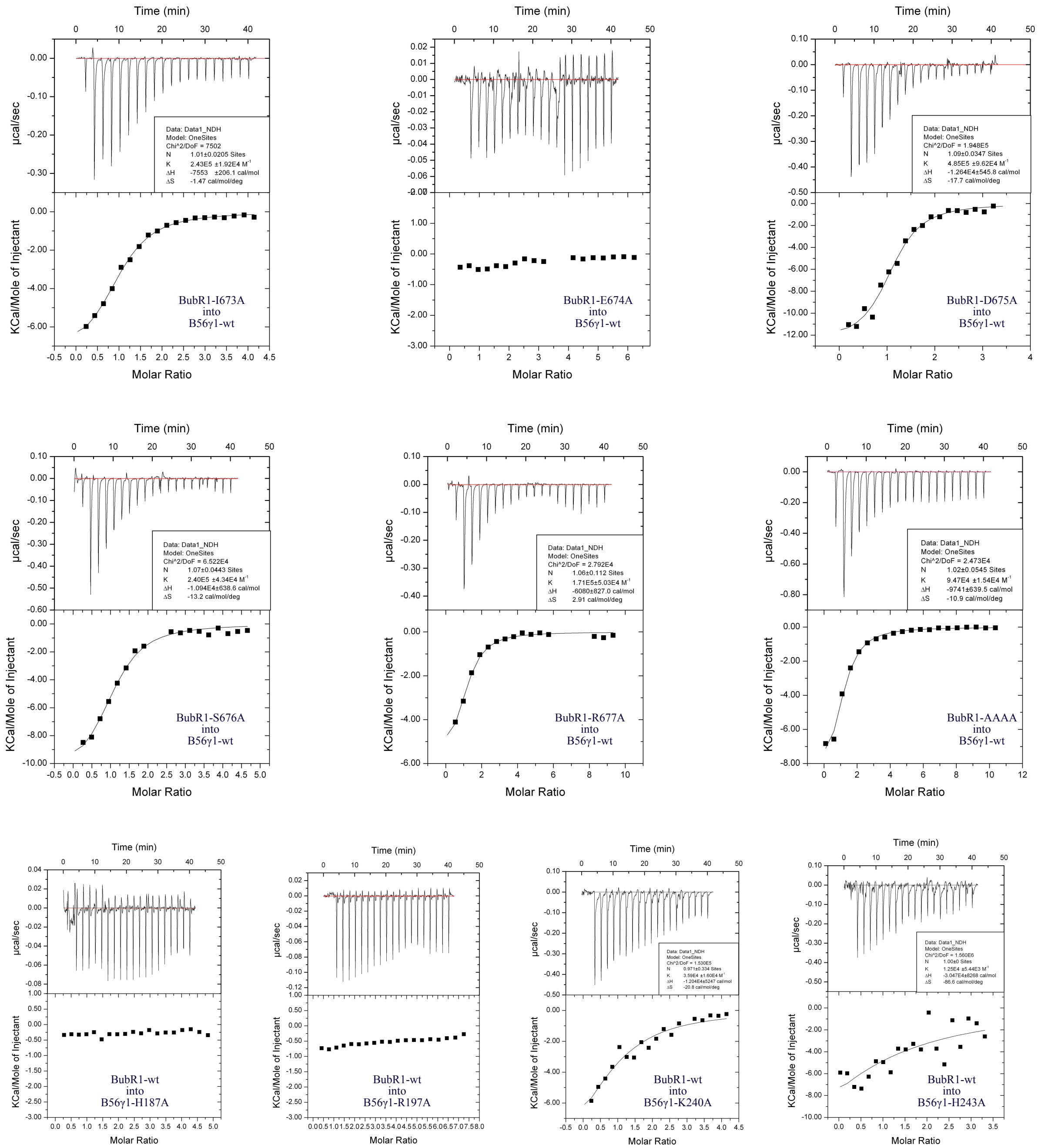

Figure S3. ITC analysis of the interaction between PP2A B56γ1(30-380) and BubR1(647-720), for both WT and mutant proteins (raw data for Table 1).

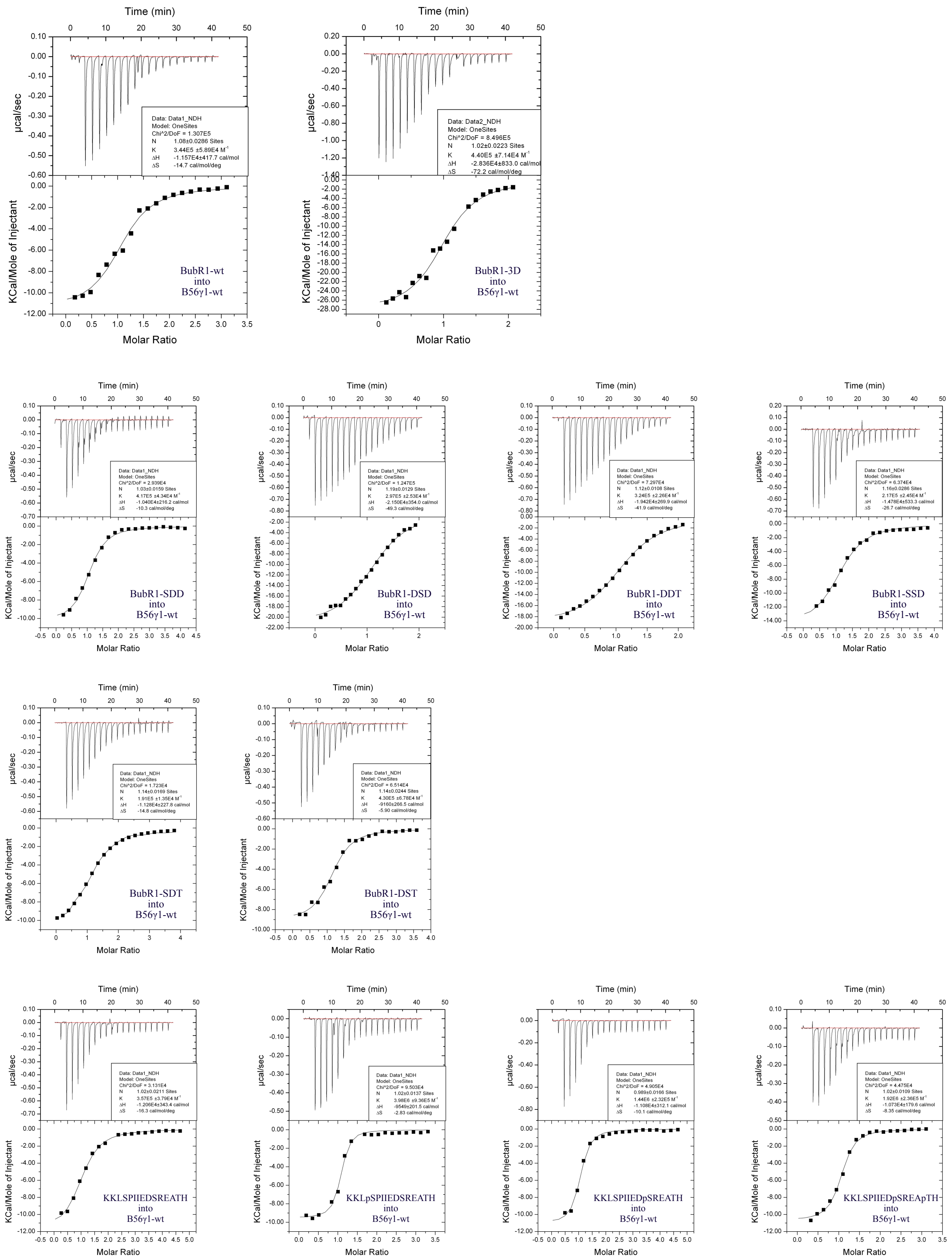

Figure S4. ITC analysis of the interactions between PP2A B56γ1(30-380) and phosphorylated or phosphor-mimicking BubR1 proteins/peptides (raw data for Table 2).

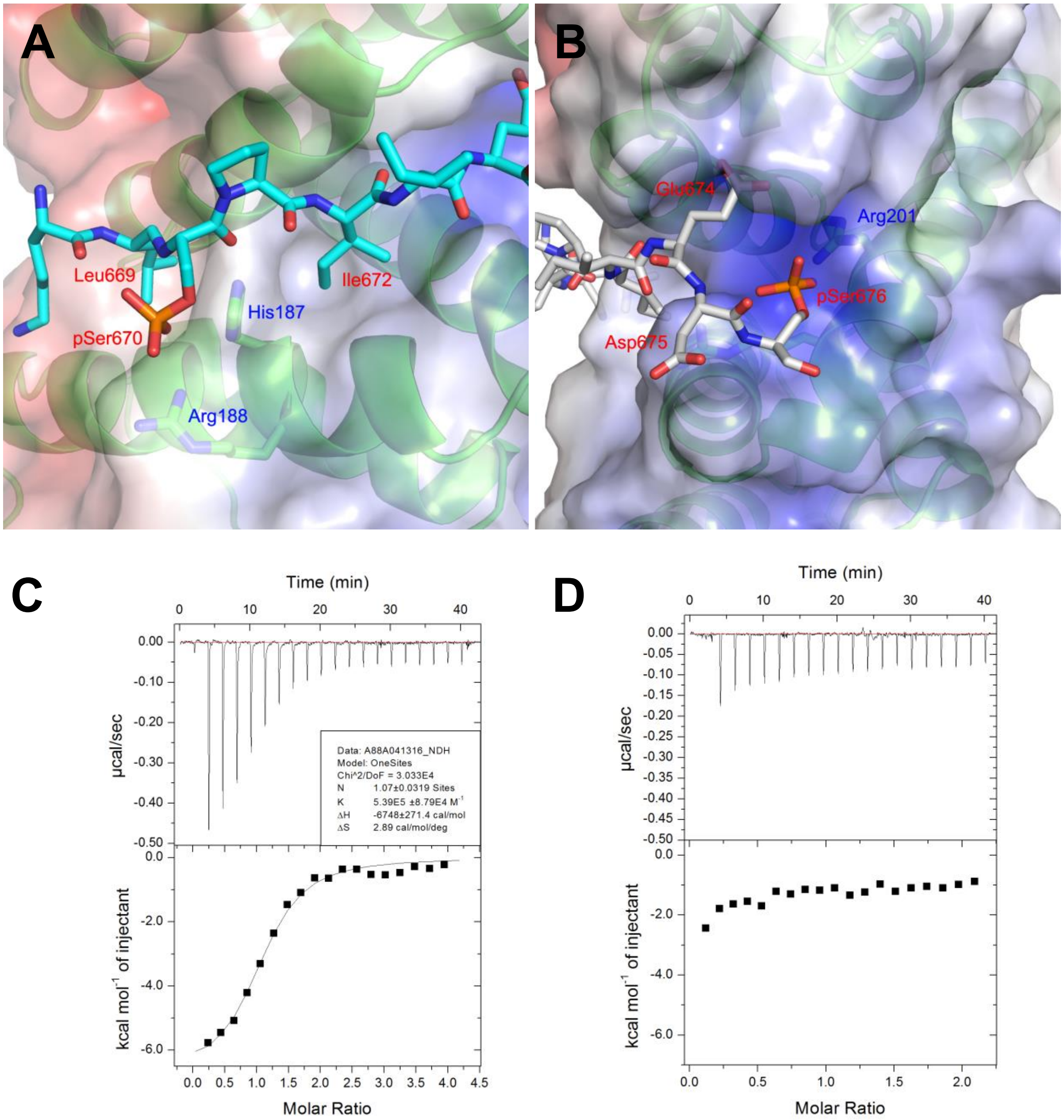

Figure S5. Models for BubR1 pSer670 and pSer676 enhanced binding. (A) Phosphorylation of BubR1 Ser670 is likely to enhance the B56-BubR1 interaction by interacting with Arg188 and His187 in the neighborhood. The Asp670 sidechain cannot mimic the phosphoserine sidechain since it is too short to reach Arg188. (B) Phosphoserine 676 of BubR1 is likely to interact with B56γ1 Arg201. (C) ITC analysis of the interaction between the B56γ1(30-380) R188A mutant and the $KKL_pSPIIEDSREATH$ phosphopeptide. (D) ITC analysis of the interaction between the B56γ1(30-380) R201A mutant and the $KKLSPIIED_pSREATH$ phosphopeptide.
